# Translating the behaviour change technique taxonomy version 1 into Spanish: Methodology and validation

**DOI:** 10.12688/wellcomeopenres.21388.1

**Published:** 2024-06-10

**Authors:** Oscar Castro, Gabriela Fajardo, Marie Johnston, Denise Laroze, Eduardo Leiva-Pinto, Oriana Figueroa, Elizabeth Corker, Jeanette A. Chacón-Candia, Giuliano Duarte

**Affiliations:** 1 Future Health Technologies, Singapore-ETH Centre, Campus for Research Excellence and Technological Enterprise (CREATE), Singapore, 138602, Singapore; 2 Centre for behaviour Change, University College London, London, WC1E 6BT, UK; 3 Department of Management, Universidad de Santiago de Chile (USACH), Santiago, 9170022, Chile; 4 Aberdeen Health Psychology Group, Aberdeen University, Aberdeen, AB24 3FX, UK; 5 Centro de Investigación en Complejidad Social, Facultad de Gobierno, Universidad del Desarrollo, Santiago, Santiago Metropolitan Region, 7590943, Chile; 6 Faculty of Human Sciences, Universidad Bernardo O’Higgins, Santiago, 8370993, Chile; 7 Department of Anthropology, Universitat Autònoma de Barcelona, Barcelona, 08193, Spain; 8 Laboratorio de Comportamiento Animal y Humano (LABCAH), Centro de Investigación en Complejidad Social (CICS), Universidad del Desarrollo, Santiago, 7610658, Chile; 9 Clinical and Applied Psychology Unit, Department of Psychology University of Sheffield, Sheffield, S10 2TN, UK; 10 Grounded Research, Rotherham Doncaster and South Humber NHS Foundation Trust, Rotherham, DN4 8QN, UK; 11 Department of Experimental Psychology, and Mind, Brain and Behaviour Research Center (CIMCYC), University of Granada, Granada, 18010, Spain; 12 Faculty of Medical Sciences, School of Obstetrics and Childcare, University of Santiago of Chile (USACH), Santiago, 9170022, Chile; 13 Faculty of Psychology, University of Barcelona, Barcelona, 08007, Spain

**Keywords:** TTsCCv1, BCTTv1, behavioural science, classification system, health, behaviour

## Abstract

**Background:**

Precise and unequivocal specification of intervention content is key to facilitating the accumulation and implementation of knowledge. The Behaviour Change Technique Taxonomy v1 (BCTTv1) is the most widely used classification of behaviour change techniques (BCTs), providing a shared, standardized vocabulary to identify the active ingredients of behavioural interventions. However, the BCTTv1 is only available in English and this hampers its broad use and adoption. The aim of the present article is to report the process of translation of the BCTTv1 into Spanish.

**Methods:**

A bilingual team led the translation of the BCTTv1, involving seven iterative steps: (i) establish a Committee, (ii) forward translation from English to Spanish, (iii) back translation from Spanish to English, (iv) comparison of original BCTTv1 and back translation, (v) opportunistic comparison against an independent BCTTv1 translation, (vi) empirical testing, and (vii) final Committee review.

**Results:**

Changes as a result of the translation process included relabelling BCTs, amending definitions, and fixing conceptual and grammatical inconsistencies, yielding the final version. Very satisfactory inter-coder reliability in BCT identification was observed as part of the empirical testing (i.e., prevalence and bias-adjusted kappa scores > 0.8).

**Conclusions:**

This work provides the Spanish-speaking population with a rigorous and validated BCTTv1 translation which can be used in both research and practice to provide a greater level of intervention detail for evidence synthesis, comparison, and replication of behaviour change interventions. The translation process described here may prove helpful to guide future translation efforts in behavioural science and beyond.

## Plain language summary

Behaviour change interventions use specific techniques (for example, providing information or setting up goals) to promote beneficial changes such as increasing physical activity or cutting down carbon emissions. Having a unifying framework for describing these techniques can help us describe studies more consistently and so better integrate research findings. The Behaviour Change Technique Taxonomy v1 (BCTTv1) is the most popular classification of intervention content. However, the BCTTv1 is only available in English and this makes it difficult for the taxonomy to be widely used as English is not the first language of so many across the world. The aim of the present article is to report the process of translation of the BCTTv1 into Spanish. A bilingual team led the translation of the BCTTv1, involving seven iterative steps: (i) establish a Committee, (ii) a first (forward) translation from English to Spanish, (iii) a second (back) translation from Spanish to English, (iv) comparison of original BCTTv1 and back translation, (v) opportunistic comparison against an independent BCTTv1 translation developed by a different research group, (vi) testing whether researchers can consistently apply the translated taxonomy, and (vii) final Committee review. Changes because of the translation process included relabelling some of the techniques, amending definitions, and fixing conceptual and grammatical inconsistencies, resulting in the final version. Results after using the final translated BCTTv1 suggest researchers can consistently apply the translated taxonomy to different behaviour change interventions. This study provides researchers and practitioners with a translation of the BCTTv1 that can be used to better specify behaviour change interventions in Spanish-speaking countries or other countries with large Spanish-speaking populations. The translation methodology described here could benefit the research community more broadly by informing future translation efforts of other research tools and resources.

## Introduction

The solution to many of the health and environmental challenges humanity faces today lies in changing people’s behaviour. To achieve this, it is crucial to effectively use and build upon evidence from behaviour change interventions. These interventions are, however, highly complex and involve many different components. This makes them challenging to replicate in research, implement in the real world, and synthesize in literature reviews ( Michie *et al*., 2011).

The Behaviour Change Technique Taxonomy version 1 (BCTTv1) was developed to provide researchers and practitioners with a tool to precisely identify Behaviour Change Techniques (BCTs). A BCT is an observable, replicable, and irreducible component of an intervention designed to alter or redirect causal processes that regulate behaviour ( Michie *et al*., 2013). The BCTTv1 was developed through a series of Delphi-type consensus exercises involving 54 experts in psychology, behavioural medicine, and health promotion ( Michie *et al*., 2013), with the ultimate aim of facilitating: (i) accurate replication of interventions (e.g., in comparative effectiveness research), (ii) faithful implementation of effective interventions, (iii) identification of most effective BCTs through systematic reviews, (iv) intervention development (drawing from a comprehensive, cross-behaviour list of BCTs), and (v) linkage of BCTs with theories of behaviour change to explore a possible mechanism of action. The taxonomy is composed of 93 consensually agreed, distinct (non-overlapping) BCTs hierarchically organised in 16 groups.

Since its development, the BCTTv1 has been extensively applied to characterise a wide range of behaviour change interventions in health and other fields ( Presseau *et al*., 2015; Scott *et al*., 2020; Weissman *et al*., 2022). The article introducing the BCTTv1 and documenting its development has been cited over 6,000 times ( Michie *et al*., 2013), and the taxonomy is widely regarded as the ‘go-to’ tool for specifying intervention content in behavioural science. However, the BCTTv1 was made available only in English. This is similar to many research-produced tools (e.g., questionnaires), as English is the lingua franca of science and various specialized fields. It is anticipated that the availability of BCTTv1 in multiple languages will increase its use and broad adoption in research, practice, and education among non-English-speaking countries, particularly low- and middle-income countries where knowledge of the English language is limited. An accurate and rigorous process of translating research tools into languages other than English is key to ensuring equity of access to health interventions in non English-speaking populations.

The objective of the present article is to describe the different stages and activities involved in the translation of the BCTTv1 into Spanish. Spanish – a Romance language that evolved from colloquial Latin spoken on the Iberian Peninsula – is the world's second-most spoken native language and the world's fourth-most spoken language overall, after English, Mandarin Chinese, and Hindustani ( Instituto Cervantes, 2019). In addition, Spanish is the official language of 20 countries in Europe and Latin America. The translation process described here may prove helpful to inform future translations of the BCTTv1 and/or other research-based tools more broadly.

## Methods

The translation process involved seven iterative phases and followed the translation and back-translation methodology ( Ozolins *et al*., 2020), whereby a translation is back-translated into the original language to allow the research team to see how closely the translation corresponds to the source material. The aim is to detect differences between the original and the back translation versions (e.g., inconsistencies or conceptual errors), which may point to problems in the actual translation and prompt further modifications ( Jakobson, 1959). Following its original use to translate psychosocial questionnaires in the 70s ( Brislin, 1970), the translation and back translation method has become a gold standard in many fields ( Behr, 2017; Chen & Boore, 2010) and has been adopted by health bodies such as the World Health Organization, which recommends this method to ensure translation equivalence ( World Health Organization, 2019). In addition to the translation and back translation method, we followed the guidance on cultural adaptation of measures in health psychology ( López-Roig & Pastor, 2017) going beyond a simple translation of words and ensuring that the culturally appropriate meaning is achieved.

### Step 1: Establish a committee to review the process of translation

At several stages in the translation process, a Committee is required to initiate and review different versions of the translation. The Committee was assembled to include: behaviour change intervention experts in both Latin America (Chile: GD, GF, DL, ELP, OF) and Spain (OC); experts in BCTs and the BCTTv1 (MJ, EC); and native speakers of Spanish (Chile: GD, GF, OF, ELP, DL; Spain: OC) and English (UK: MJ, EC; USA: DL). In addition, some of the Spanish-native speakers had a high proficiency in English (OC, GF) or were bilingual in both languages (DL).

### Step 2: Forward translation from English to Spanish

The forward translation of the original BCTTv1 (introduction, BCT labels, definitions, and examples) from English to Spanish was completed by a professional translator, bilingual in English and Spanish, independent of the Committee, and with no specific expertise in behaviour change interventions. This first translation was reviewed by the Spanish-speaking members of the Committee to clarify inconsistencies, to ensure cultural appropriateness in both Latin America and Europe, and to check that the conceptual meaning of the BCTs was maintained. Changes to the version produced by the translator were discussed and agreed upon in meetings with the whole Committee. Although slight differences exist in the Spanish language spoken in different regions, the Committee aimed to use standard terminology as much as possible to produce a translation that can be used for all Spanish-speaking countries.

### Step 3: Back translation from Spanish to English

Once the forward translation was revised and agreed upon by the Committee, it was sent to a certified, professional translator, bilingual in English and Spanish. This translator was different from the one employed for the forward translation and had no knowledge of the original English version.

### Step 4: Comparison of original BCTTv1 and back translation

The original and the resulting back-translated versions in English were compared independently by two native English speakers with expertise in BCTTv1 (MJ and EC), who identified any conceptual and grammatical inconsistencies of the back translation with the original. Discrepancies were additionally discussed by OC and MJ (providing both domain expertise and competence in both languages) to check their equivalence and identify any differences in wording and meaning. It is recognised that wording may differ, but this is only important if the ‘semantic, idiomatic, experiential or conceptual’ ( López-Roig & Pastor, 2017) meaning is altered. OC, MJ, and EC then discussed differences between the original and back-translated versions and brought any remaining issues for resolution by the full Committee. At this stage, the Spanish translation of the BCCTv1, named ‘Taxonomía de Técnicas de Cambio de Comportamiento versión 1’ or ‘TTsCCv1’, was deemed ready for empirical testing.

### Step 5: Opportunistic comparison against a parallel independent BCTTv1 translation

Before starting the empirical testing, we identified a second forward translation of the BCTTv1 into Spanish that had been recently completed by an independent team at a charitable organisation (Swiss Red Cross). This second translation was performed by a professional translator and reviewed by a European academic/healthcare worker (NR) who had training in behaviour change interventions, had no knowledge of the first translation, and was motivated to translate the BCTTv1 to facilitate her practical work; in addition, she had experience of working in different Spanish speaking countries.

After approaching the Swiss Red Cross, the Committee was granted access to use this parallel translation for comparison. Native Spanish speakers compared this version with the Committee’s translation to identify discrepancies and to check for any differences in wording and meaning. The Committee then discussed whether changes were necessary in the TTsCCv1.

### Step 6: Empirical testing (feasibility and reliability of the TTsCCv1)

Empirical testing was designed to assess the feasibility and reliability of the TTsCCv1 to identify BCTs from behaviour change intervention reports. This step is referred to as ‘pilot testing’ for the WHO-recommended translation approach ( World Health Organization, 2019). Analyses mimicked (and made use of) the reliability analyses that were conducted for the original BCTTv1 ( Abraham *et al*., 2015; Michie *et al*., 2015), with four relevant research questions posed in relation to TTsCCv1’s empirical testing:

1. How feasible is it to use the TTsCCv1 to annotate reports of behaviour change interventions? To this end, two native Spanish speakers with English proficiency (OC and JC) used the TTsCCv1 to annotate (i.e., identify BCTs) in 20 intervention descriptions from published protocols. These descriptions were selected at random from the original pool of 40 intervention descriptions used for the empirical testing of the BCTTv1, which covered a range of behavioural domains.

An annotation task booklet (comprising the 20 intervention descriptions and task instructions) was developed by OC and shared with JC. Both coders were trained in coding using BCTTv1 ( Wood *et al*., 2015) and discussed feasibility upon completing the task, including ease of using TTsCCv1, differences in annotating between the two coders, and any changes needed to clarify TTsCCv1.

2. To what extent do trained coders agree when using the TTsCCv1 to identify BCTs in behaviour change intervention reports (i.e., how good is the inter-coder reliability of the Spanish BCTTv1)? As with the original BCTTv1 validation work, inter-coder reliability was assessed using the prevalence and bias-adjusted kappa (PABAK) statistic ( Byrt *et al*., 1993). PABAK was chosen because it adjusts for both prevalence of occurrence of BCTs and bias in rates of identification of BCTs. For example, when both coders agree that many BCTs are absent and/or the chance of agreement is high ( Gwet, 2014). Regarding the acceptability of the level of agreement, Fleiss *et al*. (2013) described PABAK values > 0.7 as an expression of a good/satisfactory agreement, and values > 0.8 as a very satisfactory agreement (range 0 to 1). PABAK scores were calculated for each intervention description and averaged on an overall PABAK score.

3. How does the level of agreement by coders using the TTsCCv1 correspond to that of coders using the original (English version) BCTTv1? The overall PABAK score representing the agreement between the two coders using the TTsCCv1 (calculated as part of research question No. 2) was compared against the published inter-coder reliability using BCTTv1.

4. To what extent do trained coders using the TTsCCv1 agree with a consensus coding by taxonomy developers? This involved assessing the inter-coder reliability between (i) the two coders using the TTsCCv1 and (ii) the resolved taxonomy developer identifications for the same 20 intervention descriptions (i.e., a proxy for validity by testing against the only available type of ‘gold standard’). This was done independently for each coder. That is, two overall PABAK scores were calculated, one for the agreement between OC and taxonomy developers, and another one for the agreement between JC and taxonomy developers.

### Step 7: Final committee review

The Committee met to review all preceding steps, make any final adjustments and agree on the final TTsCCv1 before proceeding to publication.

## Results

### Step 1: Establish a committee to review the process of translation

GD led the formation of the Committee to include the full range of expertise including representation from Latin America and Spain. A member of the team (MJ) was part of the original Behaviour Change Technique Taxonomy project (2010–2013) that led to the BCTTv1. The Committee met via Zoom on 11 occasions during 2022 and 2023, with meetings conducted in both Spanish and English.

### Step 2: Forward translation from English to Spanish

In reviewing the first translation, the Committee of native Spanish speakers and behaviour change experts identified some grammatical, but especially contextual inconsistencies that could lead to errors and corrected the document. For example,

- The direct Spanish translation of “BCT” is “TCC”. However, TCC is used to denote Cognitive-Behavioral Therapy in Spanish (“Terapia Cognitivo-Conductual”). To avoid this problem the abbreviation of BCT was translated as TsCC, as “Behavioural Change Techniques” translated to Spanish is “Técnicas de Cambio de Comportamiento”.
- Another example included “coping planning”, which in the first version was translated as “planificación para enfrentar problemas” but the Committee thought “estrategias de afrontamiento de problemas” was better adjusted to the Spanish language idiomatically.
- Another adjustment from the first version was due to one word having two meanings in English. For example, the BCT label "thinning" was translated as "adelgazamiento", meaning thinning of the body rather than the behavioural concept referring to the reduction of reinforcements over time. We replaced this word with "reducción" as the nearest to the intended meaning
- For some words it was difficult to find a direct translation that retained the original meaning. For example, “encourage” is a key concept in BCTTv1 but is difficult to translate directly. After considerable searching and discussion, it was agreed to use “animar” consistently throughout the translation.

The BCTTv1’s forward translation by the professional translator is available in Supplemental Material 1 ( Laroze *et al*., 2021). The second translation version produced after the Committee’s revision in Supplemental Material 2 ( Laroze *et al*., 2021).

### Steps 3 and 4: Back translation from Spanish to English and comparison with original BCTTv1

Discussion of the back translation resulted in some linguistic and conceptual changes, for example:

- In the original version, every description started with a verb. However, in the back-translation, some of the descriptions started with nouns or adjectives. This was revised and corrected accordingly in the Spanish translation to ensure consistency.
- The word “prompt” has no unique translation in Spanish, so depending on the context different words were used. Consequently, it was also back-translated with different words that do not convey the same meaning. The Committee made the decision to stay consistent and use “Incitar” in Spanish every time that “prompt” was used in the original English version.
- The label of the BCT 2.2 was back-translated as “Behavioural feedback”, but this might mean that the feedback *is* behavioural, instead of its original meaning which is to give feedback on someone’s behaviour. In Spanish, there is very little difference between both ways of phrasing, so it only required a minor preposition change to make it clearer.
- In the original version, the label for BCT 1.7 was “Outcome goals”, but to be accurate and clear in the translation, we translated it as “Objetivos definidos como resultados”, for which its literal translation in English is “Goals defined as outcomes”.

It is worth noting that a team decision was made at this step to not back translate the BCT examples and focus on the BCTs’ labels and definitions. This was done for two main reasons: (i) the original examples in the English version are currently under a reformulation and updating process, so a translation of them would quickly become obsolete, and (ii) the contextual nature of the BCT examples, which prompts careful consideration of additional cultural and institutional elements according to the local reality. Context-specific examples would need to be developed according to the planned region of use. Dealing with these issues would have caused a major delay in the publication of this resource. The BCT examples in BCTTv1 were thus only forward translated by an independent professional translator (step 2) and then reviewed by two native Spanish speakers, two from Chile (GF, GD) and one from Spain (OC). They checked and adjusted the examples for idiomatic and experiential meanings in both cultures.

The BCTTv1’s back translation is available in Supplemental Material 3 ( Laroze *et al*., 2021). The translation version produced after the Committee’s comparison between the back translation and the original BCTTv1 is also available in Supplemental Material 4 ( Laroze *et al*., 2021).

### Step 5: Opportunistic comparison against a parallel independent BCTTv1 translation

Comparison with the independent translation mainly confirmed the results of the Committee’s translation. Every difference was discussed; a few synonyms were identified and some slight differences in nuance were resolved. Some examples of these word changes were:

- In the Committee’s translation, “environment/environmental” was translated as “ambiente/ambiental”, but the parallel version differentiated when this environment refers to the close or proximate space that someone occupies (“entorno”) and when it refers to a more general climate-related environment (“medioambiente”), since there are different words in Spanish for both instances. As such, when the original description or label refers to the first meaning, we used “entorno”, and when it refers to the second, we changed it to “medioambiente”.
- In our version, we translated “to perform” or “performance” as “realización”, but the parallel version translated them as “desempeño”. The Committee decided to keep “realización” throughout, making only one exception in the description of BCT 6.2, where we changed it to “desempeño” as it was a better fit with the rest of the sentence.
- One of our greatest challenges was to find a translation for “buddies” (part of the description of label BCT 3.1) into Spanish that worked for most Spanish-speaking countries and contexts and that retained the intended meaning. First, our team translated it as “compinches”, but in the parallel version, it was translated as “cuates”, which is a very typical word for Mexico and Guatemala. Since neither of these satisfied the cultural width and neutrality we intended in this work, we changed it to “compañeros”.

The translation version produced after the Committee’s comparison between the TTsCCv1 and parallel independent BCTTv1 translation is available in Supplemental Material 5 ( Laroze *et al*., 2021).

### Step 6: Empirical testing (feasibility and reliability of the TTsCCv1)

For the empirical testing, we guided our work by the research questions proposed in the methods section:

1. How feasible is it to use the TTsCCv1 to annotate reports of behaviour change interventions? The TTsCCv1 was deemed feasible by the two coders and no additional changes were recommended. One of the coders (JC) had a Spanish linguistic background (Mexico) different from the Committee’s background (Chile and Spain) and reported no issues understanding and using the TTsCCv1. This reinforced the idea that the taxonomy can be used by a wide range of different Spanish-speaking populations. Anecdotally, coders reported that using the tool was difficult at first because of their previous experience using the BCTTv1 and becoming used to the labels and definitions in English, but this was just temporary and possibly not a relevant barrier for TTsCCv1’s target population.

2. To what extent do trained coders agree when using the TTsCCv1 to identify BCTs in behaviour change intervention reports (i.e., how good is the inter-coder reliability of the Spanish BCTTv1)? The mean PABAK scores between OC and JC ranged from 0.78 to 0.97 (overall mean=0.89; SD=0.05; overall median=0.91) across the 20 intervention descriptions. Thus, overall, very satisfactory inter-coder reliability was observed when identifying BCTs by the two native Spanish speakers when using the TTsCCv1.

3. How does the level of agreement by coders using the TTsCCv1 correspond to that of coders using the original (English version) BCTTv1? The overall mean PABAK of the BCTTv1 (across 20 pairs of trained coders annotating 20 intervention descriptions) was 0.86 (range=0.07, SD=0.02). Therefore, the level of agreement was similar to the one achieved between the two native Spanish speakers when using the TTsCCv1 (overall mean=0.89).

4. To what extent do trained coders using the TTsCCv1 agree with a consensus coding by taxonomy developers? The mean PABAK scores between OC and the consensus coding by taxonomy developers ranged from 0.84 to 1 (overall mean=0.93; SD=0.04; overall median=0.95) across the 20 intervention descriptions. The mean PABAK scores between JC and the consensus coding by taxonomy developers ranged from 0.82 to 0.95 (overall mean=0.9; SD=0.05; overall median=0.93) across the 20 intervention descriptions. Thus, overall, very satisfactory inter-coder reliability was observed between the two native Spanish speakers and a gold-standard proxy provided by the taxonomy developers. The annotation task booklet, together with OC, JC, and the taxonomy developers’ BCT identification for each of the 20 intervention descriptions is available in Supplemental Material 6 ( Laroze *et al*., 2021).

### Step 7: Final committee review

The final TTsCCv1 was reviewed and agreed upon. As a result, we ended up with a fully revised Spanish version of the BCT Taxonomy v1, ready to be applied by practitioners and researchers (Supplemental Material 7 ( Laroze *et al*., 2021)).

## Discussion

This paper describes a systematic and iterative approach for translating the original (English) version of the BCTTv1 into Spanish. The process included seven steps, combining forward and back translation methods as well as empirical testing. By having a comprehensive, cross-behaviour, and widely accepted BCT taxonomy available in their native language, the TTsCCv1 is expected to assist Spanish-speaking populations in better designing, implementing, evaluating, and reporting behaviour change interventions for both practice and research. The work reported here is a step towards the objective of disseminating agreed methods that permit and facilitate precise reporting of complex behavioural interventions, in line with existing intervention reporting recommendations such as CONSORT ( Antes, 2010). The fact that an independent parallel translation of the BCTTv1 was identified during our translation process to facilitate the Swiss Red Cross’ practical work highlights the need and potential impact of having a rigorous published BCTTv1 translation for the Spanish-speaking world. We consider the TTsCCv1 to be relevant for the 20 predominantly Spanish-speaking countries as well as the United States or other English-speaking countries with large Spanish-speaking populations.

Moreover, we believe the methodology described here could be used to translate the BCTTv1 to other languages, further increasing the taxonomy’s widespread use and acceptance. This could also help fulfil one of the main ‘future developments’ objectives of the original taxonomy developers, which was to increase the diversity of expertise and the geographical and cultural contexts in which BCTs are used, contributing to elucidate the extent to which BCT Taxonomy v1 is relevant across contexts, countries, and cultures and the extent to which specific adaptations are needed ( Michie *et al*., 2013). In addition to the BCTTv1, our methodology could be replicated to translate other research tools more broadly, such as questionnaires, public health guidelines, or taxonomies. Since the development of the BCTTv1, other taxonomies in behavioural science have been published for specific purposes, such as a taxonomy of self-enactable techniques to change and self-manage motivation and behaviour ( Knittle *et al*., 2020), a taxonomy of BCTs for promoting shared decision making by healthcare professionals ( Agbadjé *et al*., 2020), and a taxonomy of BCTs used by affected others impacted by gambling harm ( Booth *et al*., 2021).

More recently, the BCTTv1 has been developed into an ontology, the Behaviour Change Technique Ontology (BCTO) ( Marques *et al*., 2024) as part of the Behaviour Change Intervention Ontology ( Michie *et al*., 2021). BCTO provides unique computer-readable identifiers for 247 BCTs. The original BCTs in BCTTv1 are linked directly to the BCTO, thus ensuring that the BCTs in TTsCCv1 can be directly linked to the ontology.

### Strengths and limitations of our translation methodology

A strength of our translation methodology relies on the use of a well-established forward and back translation process ( World Health Organization, 2019), together with further guidance on cross-cultural adaptations to go beyond literal translation and consider cultural meaning ( López-Roig & Pastor, 2017). It is worth noting that our approach combined professional translation support with domain-specific expertise in behaviour science. While the inclusion of translation experts in research teams is recommended ( Ozolins *et al*., 2020), the two previous translations of the BCTTv1 we have identified, to European Portuguese ( Félix *et al*., 2023) and French ( Chevance, 2019), did not engage professional translators in the process. In addition, there is no evidence of empirical testing conducted with any of these two translations. An accurate translation is key to ensuring comparability between the original and the translated version of the taxonomy, and thus we see incorporating empirical testing in the translation process and providing evidence of TTsCCv1’s reliability as a strong point of our methodology.

The composition of the Committee is also a strength as it included a range of linguistic experiences (English-native speakers, Spanish-native speakers, bilingual speakers), expertise in behavioural science (including one of the original taxonomy developers), and covered a total of three Spanish-speaking countries (Chile, Spain, and Mexico), ensuring the BCTTv1 is applicable to a wide range of Spanish-speaking populations. Last, we see as positive for TTsCCv1’s development the opportunistic comparison that was conducted with an independent parallel translation from practitioners at the Swiss Red Cross, as it allowed us to contrast our translation with a more down-to-earth, usable version and make changes accordingly.

Some limitations need also to be acknowledged. Although we conducted empirical testing of the TTsCCv1, this work was not as comprehensive as the validation methods for the original taxonomy (e.g., a higher number of coders was employed to calculate the inter-coder reliability of the BCTTv1). The Behaviour Techniques Taxonomy project was a three-year endeavour funded by the UK’s Medical Research Council. In comparison, our time, manpower, and resources were limited. We expect the translation to be an iterative process in which adjustments need to be made and tested for reliability on an ongoing basis. Another limitation lies in the fact that a specific part of the taxonomy (the BCT examples) was only forward translated and checked by two members of the team, and thus it can be argued it did not undergo the same rigorous translation process as the rest of the taxonomy. We consider new examples need to be developed for the Spanish (and any future translated versions) to ensure fit with the local context. We also anticipate further work being necessary to make full use of the newly released BCTO. Nevertheless, given the urgent need for this resource, we considered it important to make it publicly available to enable the sharing of methods needed to advance knowledge of behaviour change interventions.

## Conclusions

The TTsCCv1 can be used by researchers and practitioners to improve the specification of behaviour change interventions in Spanish-speaking countries or other countries with large Spanish-speaking populations. This translation is a step forward towards BCTTv1’s global use as a cross-domain, internationally supported taxonomy to improve the accumulation and implementation of knowledge in behavioural science. The translation process described here may prove helpful to guide future translation efforts.

## Ethics approval

Ethical approval from an institutional review board (IRB) is not applicable due to the nature of the manuscript, and thus no copy of IRB approval has been made available in the submission.

## Data Availability

Open Science Framework: Translating the BCTTv1 into Spanish https://doi.org/10.17605/OSF.IO/DZQYH ( Laroze *et al*., 2021). This project contains the following extended data: Electronic Supplementary Material 1 ( https://osf.io/tcf4j) Electronic Supplementary Material 2 ( https://osf.io/fc459) Electronic Supplementary Material 3 ( https://osf.io/kzd4t) Electronic Supplementary Material 4 ( https://osf.io/2ha97) Electronic Supplementary Material 5 ( https://osf.io/tec5v) Electronic Supplementary Material 6 ( https://osf.io/rwmj8) Electronic Supplementary Material 7 ( https://osf.io/fdh29) Data are available under the terms of the Creative Commons Attribution 4.0 International license (CC-BY 4.0).
