# Supplementary material for: Translating the behaviour change technique taxonomy version 1 into Spanish: Methodology and validation
Source: Wellcome Open Res. 2024 Jun 10;9:298. [Version 1] doi: 10.12688/wellcomeopenres.21388.1 (PMC11422758; doi:10.12688/wellcomeopenres.21388.1)
Supplement: Peer review history

# Reviewer report: Reviewer response for version 1

**DOI:** 10.21956/wellcomeopenres.23653.r93966

**Published:** 2024-09-24

**Authors:** Claudia Teran-Escobar

**Affiliations:** 1 University of Paris Nanterre, Nanterre, France

1. The translation of the BCT manual is a tool that could be widely used by field staff in Spanish-speaking countries.
2. The procedure for translation and comparison with independent translations seems to me to be relevant and well detailed. The examples in the results give a better idea of the adaptations that had to be made for Spanish.
3. The existence of the committee is also reassuring and guarantees a good proximity to the original idea and objective of the BCTS.
4. I have only a few comments on the discussion that could be strengthened in relation to the potential of this article:
  Your article could potentially enable more than just better reporting of BCTs used to improve the practices of field staff in order to better develop and implement theory- and evidence-based behavior change interventions as well as reach often neglected populations (non-WEIRD populations). In particular, the Swiss Red Cross translation enabled us to teach Paraguayan health technicians to better develop, implement and evaluate behavior change interventions:
  Teran-Escobar C *et al*.(2024 ^1^ )

Is the work clearly and accurately presented and does it cite the current literature?

Yes

If applicable, is the statistical analysis and its interpretation appropriate?

Yes

Are all the source data underlying the results available to ensure full reproducibility?

No source data required

Is the study design appropriate and is the work technically sound?

Yes

Are the conclusions drawn adequately supported by the results?

Yes

Are sufficient details of methods and analysis provided to allow replication by others?

Yes

Reviewer Expertise:

Behaviour change interventions, implementation science, planetary health

I confirm that I have read this submission and believe that I have an appropriate level of expertise to confirm that it is of an acceptable scientific standard.

## References

[1] : Can we teach an old dog new tricks? Teaching how to design, implement and evaluate behaviour change interventions to health promoters in Paraguay and Bangladesh.2024; 10.31234/osf.io/rtqjd 10.31234/osf.io/rtqjd

# Reviewer report: Reviewer response for version 1

**DOI:** 10.21956/wellcomeopenres.23653.r88053

**Published:** 2024-07-29

**Authors:** Jordi Fernandez Castro

**Affiliations:** 1 Department of Basic Developmental and Educational Psychology, Universitat Autonoma de Barcelona, Barcelona, Catalonia, Spain

This article is of an instrumental and methodological nature because it consists of the translation of the Behaviour Change Technique Taxonomy v 1 (BCTTv1) into Spanish, the result of which is called Taxonomía de Técnicas de Cambio de Comportamiento (TTCCv1).

 This objective is useful for both research and clinical practice, since the BCTTv1 is a unified framework for the nomenclature of behavioural change techniques and facilitates the training, comparison and evaluation of the various psychological interventions, even if they have different theoretical bases. The translation of the BCTTv1 into Spanish can substantially increase the mass of data that can be compared, analysed and evaluated and it can facilitate the inter-cultural studies.

 This translation has been carefully programmed, something that should be appreciated since in psychological research conducted in Spanish there is an abundance of translations of material originally in English made directly by the researchers, without contrasting, which can give rise to ambiguous concepts, confusion, misunderstandings and cultural interferences.

 A meticulous and rigorous methodology has been followed, including both forward and back translations, with bilingual psychologists and professional translators involved in the process.

 The objectives included assessing the reliability and ease of use, verifying that different users can have very similar results with the translated version, and even comparing the English and Spanish versions.

 I therefore consider it to be a relevant and useful article, methodologically correct and with interesting results, which, in my opinion, can be indexed in its present form.

 In addition, I would like to make some comments on translation in case they might be of use to the authors. It is well known that translation is a complex task that never has a linear result; different good translations can be obtained from one text. The authors justify that a term in English is not always translated with the same word in Spanish. This rigidity is not necessary; for example, the word performance can and should be translated by different words depending on the context: sport, work, art, or many others.

 The same happens with the translation of prompt, which has a very specific meaning in English that does not fit with a single word in Spanish and can be translated, depending on the context, as “incitar”, “promover”, “favorecer” or “guiar”, depending above all on the intensity and specificity of the prompt. On the other hand, prompt is a word that is used in technical jargon without being translated by Spanish speakers, such as in AI, in the audiovisual world (teleprompter) or in verbal behaviour analysis.

 On the other hand, there are concepts created in English that have been incorporated into the Spanish lexicon but adapted to Spanish grammar. For example, feedback in English is a verb but in Spanish it is a noun, in Spanish one gives feedback to someone, whereas in English one feedback to another, which is why it is necessary to introduce prepositions in Spanish. By the way, feedback is used by Spanish-speaking researchers in written texts in Spanish, but the truth is that Spanish-speakers in oral language preferentially use the Anglicism feedback, pronounced "fitbac" or “fizbà” depending on zones, perhaps because the English word it is easier to pronounce and shorter.

 All in all this is an excellent, useful and worthwhile to be indexed article.

Is the work clearly and accurately presented and does it cite the current literature?

Yes

If applicable, is the statistical analysis and its interpretation appropriate?

Yes

Are all the source data underlying the results available to ensure full reproducibility?

Yes

Is the study design appropriate and is the work technically sound?

Yes

Are the conclusions drawn adequately supported by the results?

Yes

Are sufficient details of methods and analysis provided to allow replication by others?

Yes

Reviewer Expertise:

Study of stress and adaptation processes in the face of both daily and work difficulties and their impact on health.

I confirm that I have read this submission and believe that I have an appropriate level of expertise to confirm that it is of an acceptable scientific standard.
